# Effect of diacutaneous fibrolysis on the muscular properties of gastrocnemius muscle

**DOI:** 10.1371/journal.pone.0243225

**Published:** 2020-12-09

**Authors:** Carlos López-de-Celis, Albert Pérez-Bellmunt, Elena Bueno-Gracia, Pablo Fanlo-Mazas, Carlos Antonio Zárate-Tejero, Luis Llurda-Almuzara, Aida Cadellans Arróniz, Pere Ramón Rodriguez-Rubio

**Affiliations:** 1 Departamento de Fisioterapia, Universitat Internacional de Catalunya, Barcelona, Spain; 2 Institut Universitari de Investigació en Atenció Primària (IDIAP Jordi Gol), Barcelona, Spain; 3 Departamento de Fisiatría y Enfermería, Universidad de Zaragoza, Zaragoza, Spain; Virginia Commonwealth University Department of Internal Medicine, UNITED STATES

## Abstract

Diacutaneous fibrolysis is a noninvasive technique that has been shown to be effective in the treatment of musculoskeletal disorders such as shoulder pain, lateral epicondylalgia, patellofemoral pain syndrome and carpal tunnel syndrome. However, while diacutaneous fibrolysis is applied to soft tissue, its effects on muscular properties are unknown. The purpose of the present study was to evaluate the effects of diacutaneous fibrolysis on muscle properties as measured by tensiomyography and myotonometry in asymptomatic subjects. An analytical descriptive study was performed. A single session of diacutaneous fibrolysis on the gastrocnemius muscle was applied to one limb (treated limb group) and the other limb was the control (control limb group). Subjects were assessed with tensiomyography and myotonometry before treatment (T0), after treatment (T1) and 30 minutes later (T2). The primary outcomes were tensiomyography and myotonometry variables. The treated limb group showed a statistically significant increase (p<0.05) in tensiomyography parameters. A decrease in rigidity and increase in relaxation was also observed on myotonometry at T1, with some of the effects being maintained at T2. Rigidity and relaxation at T1 were statistically significant between groups (p<0.05). A single session of diacutaneous fibrolysis to the gastrocnemius muscle of asymptomatic subjects produced immediate changes in muscle properties. These changes were maintained 30 minutes after the application of the technique.

## Introduction

Diacutaneous fibrolysis (DF) is a non-invasive technique used to treat musculoskeletal conditions which produce pain and/or movement restriction [1–5]. DF is an instrument- assisted soft tissue mobilization developed from the Cyriax deep friction massage principles. It consists of a set of metallic hooks applied to the skin which seems to allow deeper penetration and a more precise application than manual techniques, providing advantages on treatment [6]. Instrument-assisted soft tissue mobilization also seems to minimize the therapist force, but increase the force applied on the tissue [7].

DF is a common technique in clinical practice which has shown to be effective in the treatment of musculoskeletal conditions such as shoulder pain [1,2], lateral epicondylalgia [3], patellofemoral pain syndrome [5] and carpal tunnel syndrome [4]. Previous studies have shown an improvement in pain intensity [2–5], function [3–5], pain-free grip strength [3], range of motion [2], nerve conduction [4] and patellar position [5]. The DF technique has also been shown to produce an increase of the range of motion in dorsiflexion of the ankle [1–3], a reduction in passive resistance of dorsiflexion of the ankle [8–10], and a decrease in the myotendinous reflex of the triceps surae [9,10] when used in healthy subjects. A recent review and metaanalisis concluded that DF is an effective technique in combination with conventional treatment to reduce pain both in a short and long-term and to increase function in patients with musculoskeletal disfunctions [6]. However, the underlying mechanisms of these effects are unknown.

In recent years, noninvasive methods for measuring muscle properties have been developed [11]. These methods include tensiomyography (TMG) and myotonometry (MMT). Tensomiography evaluates the skeletal muscle mechanical and contractile properties by means of an external electrical stimulus of controlled intensity. It provides information on the state of fatigue, muscle activation, tone or contractile properties of the muscle [12–17]. The myotonometry (MMT) measures muscle stiffness of the muscle at rest [15,18]. By means of a mechanical impulse, it provides values for the properties of the tissue such as tone, rigidity and elasticity [19]. Both methods measure parameters that describe biomechanical muscle properties in an objective way [12,16,17,20] and have been shown to be useful in the evaluation of the musculoskeletal system both in normal and pathological states [15,18,19,21–23]. Because DF is applied to soft tissue, it is assumed to modify the muscle properties [8,9]. However, no studies to date have evaluated the effect of DF on muscle properties. Therefore, in order to better understand the effects of DF, we aimed to evaluate the effects of diacutaneous fibrolysis on muscle properties as measured by tensiomyography and myotonometry in asymptomatic subjects.

## Methods

### Study design

An analytical descriptive study was conducted at the research laboratory of *Universitat Internacional de Catalunya*. The local ethics committee of *Universitat Internacional de Catalunya*–CER (*Comitè Ètic de Recerca*) approved the study protocol (study Code: CBAS-2018-18) and the study was conducted in accordance with the declaration of Helsinki [24].

### Sample

Healthy subjects aged over 18 years were invited to participate in the study. Exclusion criteria for the participation were: concomitant pathologies; infiltration in the treated area 3 months previously; language limitations that made it difficult to give informed consent; and specific contraindications to DF (skin lesions, vascular abnormalities, treatment with antiplatelet agents, acute inflammatory process). Written informed consent was obtained from each subject prior to study participation.

Since there were no previous data on the variables studied, a sample of 32 subjects was collected, similar to that in the study by Veszely et al. (2000) [9] on myotendinous reflexes in the triceps surae.

Diacutaneous fibrolysis was applied to one of the lower extremities of the subjects (treated limb group). The other extremity received no intervention (control limb group). The side to be treated was randomly assigned using a random number table (Random.org).

### Data collection, variables and measurements

The intervention and measurements were carried out by two therapists. The first therapist applied the DF. The second therapist, blinded to the group assignment of the lower extremity, took the measurements and recorded the data. To minimize bias, participants were not informed of any of the results obtained during measurement. The TMG and MMT of the gastrocnemius muscle were measured at the beginning of study (T0), after the intervention (T1) and 30 minutes after the intervention (T2).

Measurements were performed in a prone position on a padded bench with a foam pad placed just above the ankle which supported around five degrees of knee flexion. The thickest point of the gastrocnemius muscle was selected by palpation. Once identified, this position was marked with a permanent marker to ensure the sensor was placed in the same position on subsequent measurements.

**Fig 1:**
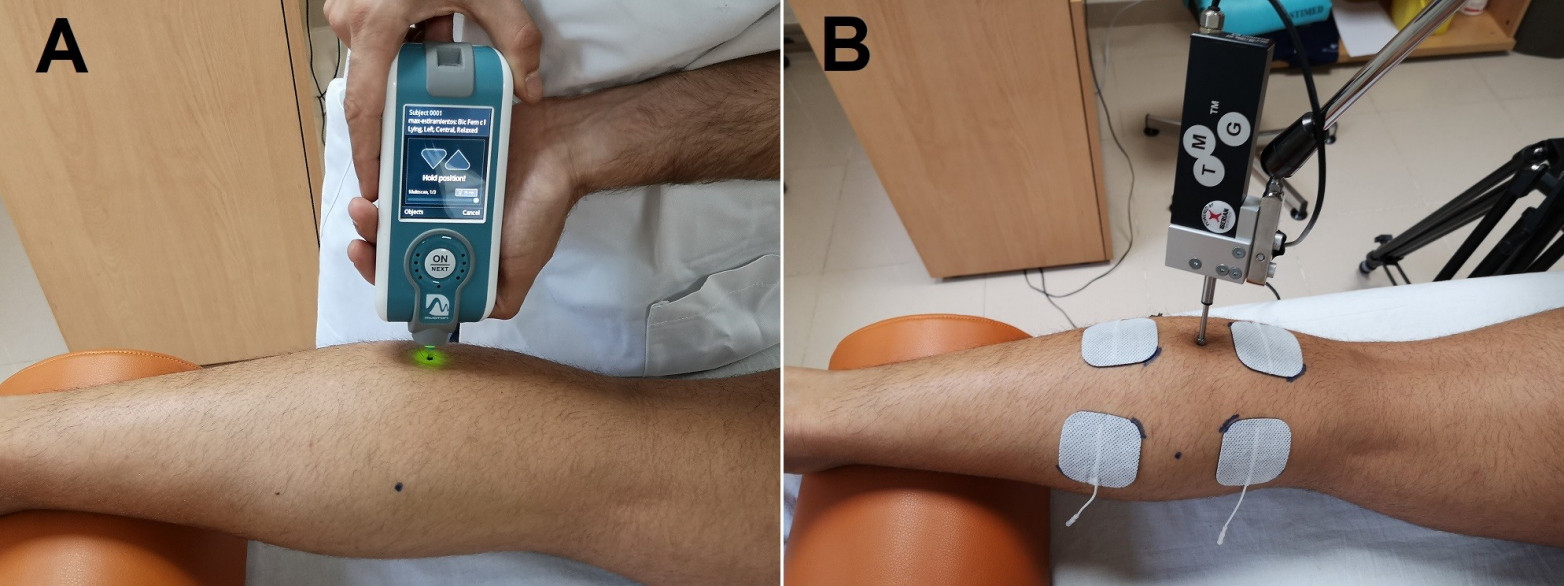
Measurement procedures. A: Myotonometry; B: Tensiomyography.

MMT was measured using the MyotonPro (MyotonPro, Myoton Ltd., Estonia), which has a good to excellent reliability (intraclass correlation coefficient [ICC] = 0.80–0.93) in healthy and clinical populations [25–30]. The probe at the end of the device was placed perpendicular to the surface of the skin overlying the gastrocnemius muscle (Fig 1A). Slight pressure was applied between the probe and the surface of the skin, and a short mechanical impulse (0.4 N for 15 ms), with a constant pre-compression force of 0.18 N, was delivered to the tissue directly under the probe. Three single measurements with a recording interval of 1 second were performed and mean stiffness values were used for data analysis. Stiffness (N/m) was calculated by the MyotonPRO system based on the equation: S = α_max_ m probe/Δl (m = the mass of the testing end, α_max_ = maximum deformation acceleration of the tissue, Δl = maximum tissue displacement) [31].

Tensiomyography was measured using a tensiomyograph (TMG-BMC d.o.o., Ljubljana, Slovenia). TMG has a good inter-observer, intra-session and between-day reliability for lower limb muscles [17,32]. TMG involves a portable device that, applied percutaneously, produces an electrical stimulus that elicits a muscular contraction, in turn detected by a digital transducer applied over the muscle belly [33] (Fig 1B). Radial muscle displacement was measured perpendicular to the muscle belly with the digital transducer Dc–Dc Trans-Tek® (GK 40, Panoptik d.o.o., Ljubliana, Slovenia). The self-adhesive electrodes (TMG electrodes, TMG-BMC d.o.o. Ljubljana, Slovenia) were placed equidistant to the measuring point, proximal (anode) and distal (cathode) to the sensor. Electrical stimulation was applied through a TMG-100 System electrostimulator (TMG-BMC d.o.o., Ljubljana, Slovenia) with a pulse of 1 ms and initial amplitude of 50 mA. For each test, amplitude was progressively increased in 10 mA increments until there was no further increase of radial displacement or maximal stimulator output (110 mA). The parameters obtained on TMG are all based on the maximal displacement (*D*m), which is the radial movement of the muscle belly after the application of the electrical stimulus, expressed in mm. The rest of the parameters obtained with TMG depend on *D*m: the delay time (*T*d), also known as reaction or activation time, is the time between the initiation and 10% of *D*m; the contraction time (*T*c) is the time between 10% and 90% of *D*m; the sustained time (*T*s) is the time in which the muscle response remains >50% of *D*m; and the half-relaxation time (*T*r) is the time in which the muscle response decreases from 90% to 50% of *D*m.

### Intervention

**Fig 2:**
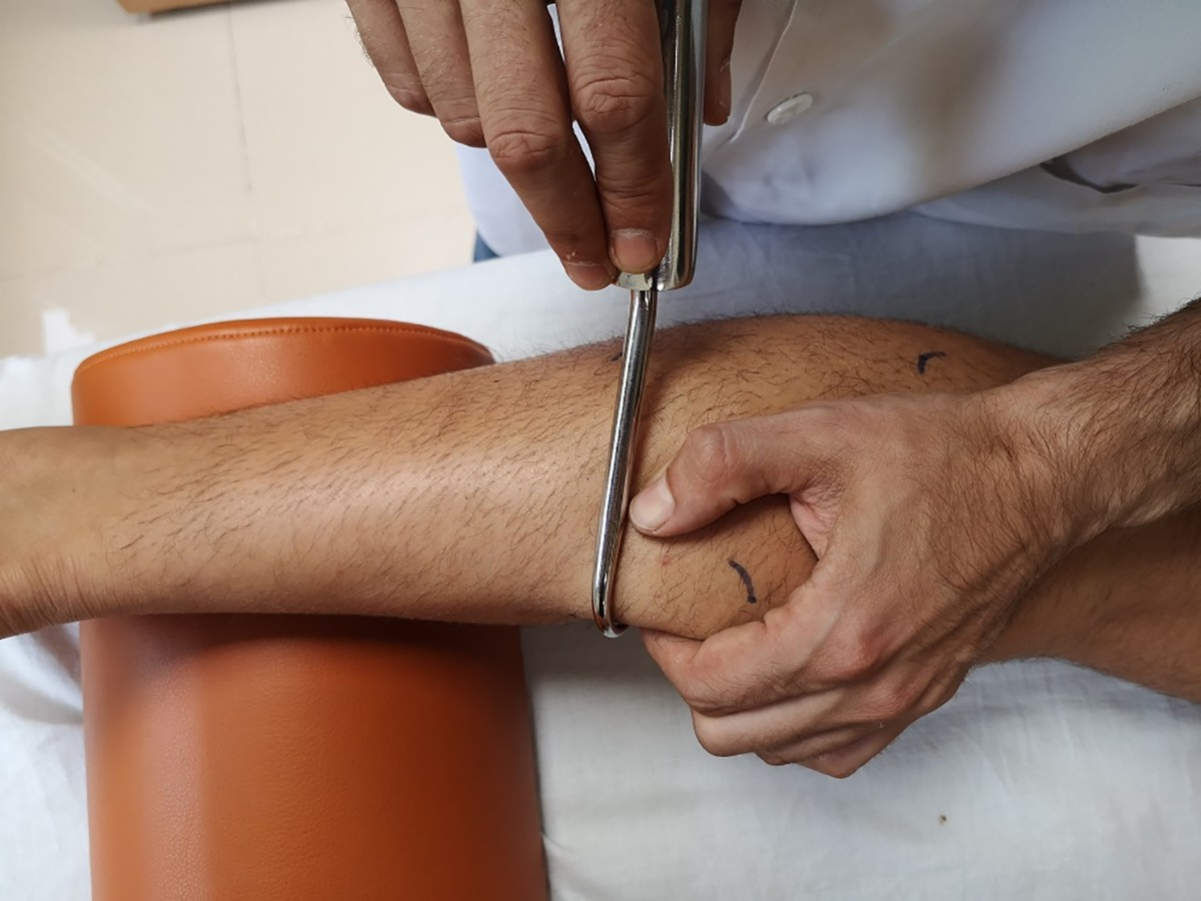
Application of diacutaneous fibrolysis on gastrocnemius muscle.

All subjects received 10 minutes of DF to the gastrocnemius muscle in the intervention limb (Fig 2). The hook was applied with the pressure required to encompass the structure to be moved, and a short fast traction was applied in a transverse direction while the hook remained fixed on the skin and underlying soft tissues. No lotion was used because DF is a safe and well-tolerated technique with no adverse effects other than mild cutaneous erythema in some subjects [1].

Participants remained lying down comfortably in a temperature-controlled room at 22°C–23°C to avoid altering muscle mechanical properties due to external factors [34]. Subjects were instructed to come for measurements in the following conditions [17]: (1) resting, with no strenuous exercise in the previous 48 h; (2) no intake of energy drinks or supplements in the previous 48 h (no alcohol or caffeine at least 3 h before measurements) and (3) no food intake at least 2 h before measurements.

Subjects were positioned prone. DF application was begun at the myotendinous junction of the medial and lateral gastrocnemius. It was continued to the intermuscular septa between the lateral gastrocnemius and soleus, soleus and peroneus muscles, medial gastrocnemius and soleus, and flexor hallucis longus tendon and the medial aspect of the Achilles tendon. It was finished with a instrumental friction (“scratching” technique) directly to the calcaneus (at the site of Achilles tendon insertion). The “scratching” technique consists of a instrumental friction applied with the hook on the tendinous insertion, similar to the Cyriax deep friction massage.

### Data analysis

IBM SPSS statistics software, version 20.0 for windows was used for all statistical analyzes. Descriptive statistics were calculated for all variables. Qualitative variables are expressed as counts and percentages. Quantitative variables and their differences are expressed as mean, standard deviation (SD) and 95% confidence intervals (95%CI). The initial homogeneity between the groups was analyzed using a paired t test or Wilcoxon test for quantitative variables.

The assumption of normality was assessed using the Shapiro–Wilk test. The intra-group comparison was performed with repeated measures ANOVA and Bonferroni post hoc test or Freidman test with Wilcoxon post hoc test. In the between-group comparisons, a paired t test or Wilcoxon test was used. A p value <0.05 was considered significant.

## Results

Of the 32 healthy volunteers recruited for the study, 15 were female (46.9%) and 17 were male (53.1%), with a mean age of 23.72 years (SD 5.18). The mean weight was 69.9 kg (SD 15.22), with a height of 174.2 cm (SD 10.9) and a body mass index of 22.5 (SD 3.01). The initial values of the outcome variables were homogeneous between the intervention and control limb groups (Table 1).

**Table 1:** Outcome variable values, intra-group analysis.

		T0	T1			T2		
		Baseline	End of treatment	Difference from baseline		30’ Post-treatment	Difference from baseline	
		Mean ± SD	Mean ± SD	Mean (95% CI)	p-value	Mean ± SD	Mean (95% CI)	p-value
**Treated Limb**	Tc (ms)	30.02 ± 9.92	39.75 ± 12.73	10.17 (5.07/15.28)	0.001	37.67 ± 12.90	8.06 (2.61/13.52)	0.002
	Td (ms)	21.35 ± 1.85	22.77 ± 1.77	1.46 (0.83/2.09)	0.001	23.28 ± 2.11	2.02 (1.22/2.83)	0.001
	Tr (ms)	61.06 ± 19.42	64.05 ± 29.63	3.99 (-9.97/17.95)	n.s.	63.29 ± 24.64	3.28 (-7.99/14.56)	n.s.
	Dm (ms)	4.40 ± 1.73	4.82 ± 1.73	0.48 (0.08/0.88)	0.014	4.75 ± 1.60	0.43 (-0.08/0.94)	n.s.
	Ts (ms)	220.52 ± 54.91	227.81 ± 49.91	6.06 (-12.60/24.73)	n.s.	236.86 ± 42.60	15.17 (-11.54/41.89)	n.s.
	Tone	14.97 ± 1.42	14.52 ± 1.23	-0.45 (-0.68/-0.23)	0.001	14.67 ± 1.30	-0.30 (-0.58/-0.03)	0.028
	Stiffness	257.89 ± 30.01	248.17 ± 26.20	-9.72 (-13.90/-5.72)	0.001	250.17 ± 26.25	-7.72 (-12.49/-2.95)	0.001
	Relaxation	21.90 ± 2.60	22.45 ± 2.23	0.55 (0.05/1.04)	0.026	22.08 ± 2.11	0.18 (-0.37/0.73)	n.s.
**Untreated Limb**	Tc (ms)	31.10 ± 11.21	34.63 ± 13.16	3.91 (-0.66/8.49)	n.s.	37.49 ± 11.62	6.84 (1.58/12.09)	0.008
	Td (ms)	22.98 ± 6.65	22.89 ± 2.28	0.00 (-3.11/3.11)	n.s.	23.26 ± 2.49	0.38 (-2.25/2.98)	n.s.
	Tr (ms)	56.81 ± 25.09	61.96 ± 30.65	6.01 (-8.77/20.78)	n.s.	68.93 ± 30.25	12.42 (-2.61/27.44)	n.s.
	Dm (ms)	4.37 ± 1.59	4.62 ± 1.46	0.30 (-0.19/0.80)	n.s.	4.75 ± 1.32	0.43 (-0.05/0.91)	n.s.
	Ts (ms)	204.32 ± 42.20	226.80 ± 55.60	21.67 (-0.22/43.56)	n.s.	230.26 ± 35.47	25.02 (10.69/39.34)	0.001
	Tone	15.03 ± 1.51	14.74 ± 1.52	-0.29 (-0.64/0.54)	n.s.*	14.72 ± 1.48	-0.02 (-0.65/0.02)	n.s.*
	Stiffness	257.05 ± 30.60	255.56 ± 27.34	-1.48 (-5.28/2.31)	n.s.	252.91 ± 27.47	-4.14 (-9.62/1.34)	n.s.
	Relaxation	21.73 ± 2.39	21.92 ± 2.12	0.19 (-0.20/0.57)	n.s.	21.85 ± 2.28	0.12 (-0.430/0.67)	n.s.

Abbreviations: n.s., not significant; SD, standard deviation. Tc, contraction time; Td, delay time; Tr, half-relaxation time; Dm, maximal displacement; Ts, sustained time; p-value: Repeated measures ANOVA and Bonferroni post hoc test.

* p-value: Friedman test and Wilcoxon post hoc test.

In the intra-group comparison, a significant increase (p<0.05) in the TMG values was observed at T1 (Tc, 10.17 ms [SD 11.20]; Td, 1.46 ms [SD 1.38] and Dm, 0.48 mm [SD 0.89]). An improvement of 8.06 ms (SD 11.97) in Tc and 2.02 ms (SD 1.76) in Td occurred at T2 (Table 1).

In the neuromuscular evaluation with MMT, only the treated limbs reached a statistically significant difference (p<0.05) at T1, showing a decrease in tone of 0.45 Hz (SD 0.50) and in stiffness of 9.72 N/m (SD 8.90) and an increase in relaxation of 0.55 ms (SD 1.11) (p<0.05). At T2, a decrease in tone of 0.30 Hz (SD 0.62) and in stiffness of 7.72 N/m (SD 10.66) (p<0.05) was observed. No statistically significant difference was found in the rest of the variables (Table 1).

In the control limbs, the only statistically significant difference (p<0.05) observed was at T2 on evaluation with the TMG: an increase in Tc of 6.84 ms (SD 11.55) and in Ts of 25.02 ms (SD 31.45). In the rest of the values at T1 and T2 measured with TMG and MMT, no statistically significant difference was observed (Table 1).

In the between-group analysis, a statistically significant difference was observed at T1 in the neuromuscular values on TMG (p<0.05) for the variable Tc 6.27 ms (SD 13.82). In addition, stiffness decreased 8.23 N/m (SD 7.56) and relaxation increased 0.36 ms (SD 0.90), reaching statistical significance (p<0.05). At T2, there was no statistically significant difference between groups for any variable (Table 2).

**Table 2:** Outcome variable values, inter-group analysis: Treated limb vs untreated limb.

	T1		T2	
	Difference from baseline		Difference from baseline	
	Mean (95%CI)	p-value	Mean (95% CI)	p-value
**Tc (ms)**	6.27 (1.19/11.33)	0.017	1.23 (-3.69/6.15)	n.s.
**Td (ms)**	1.47 (-0.99/3.93)	n.s.	1.66–0.71/4.02)	n.s.
**Tr (ms)**	-2.02 (-20.57/16.53)	n.s.	-9.13 (-21.99/3.73)	n.s.
**Dm (ms)**	0.18 (-0.29/0.65)	n.s.	-0.01 (-0.40/0.39)	n.s.
**Ts (ms)**	-15.61 (-37.08/5.87)	n.s.	-9.85 (-34.07/14.37)	n.s.
**Tone**	-0.16 (-0.44/0.12)	n.s.	0.01 (-0.24/0.27)	n.s.
**Stiffness**	-8.23 (-10.96/-5.51)	0.001	-3.58 (-7.27/0.11)	n.s.
**Relaxation**	0.36 (0.03/0.69)	0.032	0.06 (-0.36/0.48)	n.s.

Abbreviations: n.s., not significant; SD, standard deviation; Tc, contraction time; Td, delay time; Tr, half-relaxation time; Dm, maximal displacement; Ts, sustained time; p-value: Paired t test.

* p-value with Wilcoxon test.

## Discussion

To the best of our knowledge, this is the first study to examine the effect of FD on muscle properties. The results of the present study showed changes in multiple parameters of neuromuscular function after the application of DF to the gastrocnemius muscle suggesting that one application of the instrumental technique is able to modify muscle performance.

At the beginning of the study, all variables were homogeneous for both extremities but only the treated limb showed an improvement in TMG variables both at T1 and T2. An increase in relaxation and a reduction in tone and in stiffness was also observed in MMT measurements. In the control limbs, statistically significant differences were observed in Tc and Ts at T2, probably due to the muscular inactivity during 30 minutes.

Previous studies have proposed that an increase in muscle stiffness (reduced Dm) could be a predisposing factor to muscular injury [35–37]. In the present study, a reduction in muscle stiffness after the application of FD was observed. This finding could indicate the potential benefits of the DF technique for both the treatment and prevention of muscular injuries. However, it has been also suggested that a reduction in muscle stiffness could lead to a loss of muscular strength, decreasing the contraction velocity (increase in Tc) [16]. In the present study, strength was not assessed and could be interesting for future studies to assess if there is a reduction in strength after the application of DF [16]. According to existing literature, the mean reference value for the variable Dm in asymptomatic population is 8.17 mm [12]. Lower Dm values indicate a high muscular tone and stiffness, while higher Dm values indicate a lack of muscular tone or high level of fatigue [18,20,38]. The initial Dm values observed in the present study were lower than the reference value for asymptomatic population. This could be related to the demographic characteristics of the sample (sporty young people) but also to the tonic component of the triceps surae muscle. The increase in Dm after the application of DF was in concordance to the reduction in tone and stiffness observed on MMT evaluation. Previous studies have shown an increase in ankle dorsiflexion ROM and a reduction in the passive resistance of this movement after the application of the DF technique in healthy subjects [8–10]. Also, a decrease in the myotendinous reflex of triceps surae after the application of DF has been previously described [9,10]. However, the underlying mechanisms of these effects are unknown. The results obtained in the present study could suggest that they could be related to a reduction both in muscle tone and stiffness after the application of the technique.

The variables Tr and Ts showed no statistically significant differences at T1 and T2 for both groups. Some authors have reported low reliability [27] and high measurement error of the variable Tr [39–41], and have suggested not using this variables for muscle measurements [42]. Lohr et al. [14] compared the measurements of TMG and MMT in paravertebral muscles and concluded that MMT evaluation may be more reliable than TMG evaluation, and also that the changes observed in tone and stiffness throughout the study were more stable than the Tc, Td and Dm values. In the present study, a significant improvement in tone, stiffness and relaxation was observed in MMT values both at T1 and T2.

The results of the present study suggest that DF produces changes in muscle properties which could improve neuromuscular function in healthy subjects. DF could be more effective in patients with ankle limitation or deficit. However, changes in the neuromuscular response in patients has not been yet analyzed. If the effects of the FD in patients were similar to the obtained in asymptomatic subjects, it could explain the clinical benefits obtained in previous studies after the application of the FD technique [2–5]. Future studies are required to assess this.

### Limitations

The present study has some limitations. First, the long term effects of the DF technique on neuromuscular function were not evaluated. Second, the effect of a simulated DF technique was not evaluated in the present study. The superficial cutaneous stimulation of the hooks has been used in previous clinical trials and showed some beneficial effects on patients. The simulated FD technique is more superficial than the real technique [1]. Although it was not the main objective of the study, it would be interesting to evaluate the effect of the superficial cutaneous stimulation of the hooks in comparison to the real technique. Due to the design of the present study, this simulated DF technique could not be carried out.

## Conclusion

A single session of diacutaneous fibrolysis produced immediate changes, which were maintained 30 minutes after treatment, in the neuromuscular response on tensiomyography and myotonometry (decrease in tone and stiffness) of the triceps surae muscle in asymptomatic subjects.

## Supporting information

S1 File (XLSX)
